# Salpingitis in Non-Sexually Active Girls: Clinical Spectrum and Diagnostic Clues from a Pediatric Cohort

**DOI:** 10.3390/children13030311

**Published:** 2026-02-24

**Authors:** Matteo Cerutti, Marta Verzieri, Lisa Gamalero, Erica Bencini, Ilaria Brizzi, Gaia Varriale, Stefano Stagi, Teresa Giani

**Affiliations:** 1 Pediatric Diabetology and Endocrinology Unit, Meyer Children’s Hospital IRCCS, 50139 Florence, Italy; 2 Department of Health Sciences, University of Florence, 50139 Florence, Italy; 3 Paediatric Division, ULSS2 Marca Trevigiana, Conegliano Hospital, 31015 Conegliano, Italy; 4 Pediatric Gynecology Unit, Meyer Children’s Hospital IRCCS, 50139 Florence, Italy; 5 Department of Pediatrics, Meyer Children’s Hospital IRCCS, 50139 Florence, Italy; 6 Reumatology Unit, Department of Pediatrics, Meyer Children’s Hospital IRCCS, 50139 Florence, Italy

**Keywords:** hydrosalpinx, pediatric, pyosalpinx, salpingitis, tubo-ovarian abscess

## Abstract

**Highlights:**

**What are the main findings?**

- Pediatric salpingitis in non-sexually active girls shows distinct clinical features compared with sexually active adolescents and adults, presenting predominantly with non-specific abdominal symptoms and often lacking genitourinary signs or identifiable sexually transmitted pathogens, which contribute to diagnostic delay.
- Gastrointestinal, appendiceal, and postsurgical factors, rather than sexually transmitted infections, represent the main predisposing pathways, with enteric and anaerobic organisms being the most frequently identified pathogens.

**What are the implications of the main findings?**

- Increased clinical awareness and early imaging, particularly ultrasound, are crucial to identify salpingitis in children presenting with unexplained abdominal pain and to avoid missed or delayed diagnosis.
- Improved recognition of non-sexually transmitted pediatric salpingitis may reduce unnecessary surgical interventions, optimize antimicrobial management, and potentially limit long-term reproductive sequelae.

**Abstract:**

Background: Pediatric salpingitis is rare and often underrecognized, especially in non-sexually active girls in whom symptoms are non-specific and sexually transmitted infections are absent. Delayed diagnosis may increase the risk of complications. We aimed to characterize the clinical presentation, diagnostic features, management, and outcomes of pediatric salpingitis and to identify predisposing factors in non-sexually active pediatric patients. Methods: We retrospectively reviewed pediatric cases of radiologically or surgically confirmed salpingitis at a tertiary children’s hospital (2000–2025) and conducted a narrative review of published pediatric cases. Results: Ten non-sexually active girls were included (median age 12.8 years). Abdominal pain was the most common symptom (80%), followed by fever and gastrointestinal complaints (50% and 30%, respectively); two patients (20%) were asymptomatic. Hydrosalpinx or pyosalpinx was detected on ultrasound in 80%. A causative organism was identified in 30%, predominantly enteric or anaerobic flora. All patients received broad-spectrum intravenous antibiotics; half required procedural or surgical intervention. Clinical outcomes were favorable in all cases. The literature review identified 56 additional non-sexually active girls, most of whom were postmenarchal. Abdominal pain was the predominant presentation, and gastrointestinal or anatomical predisposing factors were common. Conclusions: Non-sexually transmitted salpingitis is an uncommon but clinically relevant condition in children. Its atypical and often subtle presentation in non-sexually active girls warrants heightened clinical awareness. Early imaging and attention to gastrointestinal or postsurgical antecedents can facilitate timely diagnosis. Further multicenter studies are needed to establish diagnostic criteria and clarify long-term reproductive outcomes.

## 1. Introduction

Salpingitis is defined as acute inflammation of the fallopian tubes [1,2,3]. It is now considered part of the broader spectrum of Pelvic Inflammatory Disease (PID), which includes infections of the upper female reproductive tract and may involve the endometrium (endometritis), ovaries (oophoritis), or pelvic peritoneum (peritonitis) [1]. Different forms of salpingitis can be identified. Hydrosalpinx results from distal tubal obstruction with serous fluid accumulation, often secondary to infection, adhesions, or congenital anomalies [2,3]. Pyosalpinx is characterized by purulent collection due to persistent infection and may progress to a tubo-ovarian abscess (TOA) [4,5].

Globally, updated data on PID prevalence remain limited [6], and estimating the incidence of salpingitis independently of PID is difficult, even in adults. In many high-income countries, PID incidence declined in the early 2000s, but recent evidence suggests a plateau or even an increase [7]. In the US, 4.4% of sexually experienced women aged 18–44 and 10% of those with prior STIs (sexually transmitted infections) report a PID diagnosis [8]; in the UK, the clinical PID rate is 281 per 100,000 per year [9]. Pediatric epidemiological data are largely lacking.

In children, salpingitis represents a significant clinical challenge due to its rarity, absence of sexual exposure source, non-specific symptoms, and potential for serious long-term sequelae.

STIs are the main cause of PID in adults, with >85% of cases linked to *Chlamydia trachomatis* and *Neisseria gonorrhoeae* [1,10,11]. According to the CDC’s 2022 STI Surveillance Report, nearly two-thirds of new chlamydial infections and half of new gonorrheal infections occur in individuals aged 15–24 [12]; 10–15% of infected females develop PID [13].

In non-sexually active patients, different pathogens are implicated. PID is often polymicrobial [14,15], and bacteria such as anaerobes, enteric organisms, and those associated with bacterial vaginosis are increasingly recognized [16,17]. Among premenarchal girls, reported pathogens include group A and B Streptococci, *Escherichia coli*, *Klebsiella* spp., *Proteus mirabilis*, *Haemophilus* spp., *Bacteroides*, *Peptococcus*, *Haemophilus influenzae*, and *Streptococcus pyogenes* [2].

Complications include abscesses, chronic pelvic pain, tubal scarring, infertility, and ectopic pregnancy, all of which significantly affect reproductive health and quality of life [4]. While diagnosis may be more straightforward in sexually active patients, especially those with known STIs, multiple partners, or inconsistent condom use, it is much more difficult in the pediatric population. Symptoms are often vague and may include only abdominal pain, fever, or nausea.

While the adult literature on salpingitis is extensive [18], data in pediatric populations are limited, and systematic evidence on salpingitis in non-sexually active girls is lacking beyond sporadic case reports. To address this gap, this study describes a retrospective cohort of non-sexually active pediatric patients with salpingitis and, in parallel, presents a targeted literature review of reported pediatric cases. The primary objective was to characterize the clinical spectrum and diagnostic features of radiologically or surgically confirmed salpingitis in non-sexually active pediatric girls. Secondary objectives were to describe management strategies and short-term outcomes, to explore potential predisposing or associated conditions in this population, and to contextualize our cohort through a narrative review of published pediatric cases of non-sexually transmitted salpingitis, comparing key clinical, radiological, microbiological, therapeutic, and outcome characteristics.

## 2. Materials and Methods

We retrospectively reviewed the medical records of children and adolescents admitted to the Pediatric Department of Meyer Children’s Hospital IRCCS in Florence, Italy, with a diagnosis of salpingitis between January 2000 and June 2025. Potential cases were identified through the institutional administrative discharge database, which adopted the International Classification of Diseases-9 (ICD-9) [19]. We queried the following code labels: “salpingitis and oophoritis,” “female pelvic inflammatory disease,” “inflammatory disorders of the uterus, excluding cervix,” “cervicitis,” “pelvic peritoneal adhesions of unknown or combined origin,” and “peritonitis”.

All candidate charts were manually reviewed. Minimum diagnostic criteria for inclusion were radiological or surgical confirmation of tubal involvement. Radiological confirmation required imaging (ultrasound as first-line; magnetic resonance imaging, MRI, or computed tomography, CT, when clinically indicated) showing at least one of the following findings: (i) a dilated tubular adnexal structure compatible with hydrosalpinx; (ii) complex/echogenic intratubal content compatible with pyosalpinx; (iii) a tubo-ovarian complex or TOA; or (iv) a thickened, hyperemic and/or contrast-enhancing tubal wall.

Surgical confirmation was defined as direct visualization of an inflamed, dilated and/or purulent tube consistent with salpingitis at laparoscopy/laparotomy, with or without drainage or salpingotomy/salpingectomy. Exclusion criteria were the absence of imaging or intraoperative evidence supporting salpingitis. Sexually active girls were excluded, in line with the study objective of describing salpingitis in non-sexually active pediatric girls. For each included case, we collected demographic information, medical and surgical history, clinical presentation, laboratory and imaging findings, microbiological results, treatments (including antibiotics and procedures/surgery), and follow-up data. This study was conducted on anonymized retrospective data. According to Italian regulations and institutional policies, ethics committee approval was not required for retrospective studies using fully anonymized data. Informed consent for the use of data for research purposes had been obtained from parents or legal guardians at the time of admission as part of standard hospital procedure. Data were collected and managed using Microsoft Excel (Microsoft Corporation, Redmond, WA, USA).

We also performed a narrative review of the literature on pediatric salpingitis. We searched MEDLINE (via PubMed), EMBASE, CINAHL, and CENTRAL databases, covering all publications up to 30 June 2025. A complementary manual search was conducted using Google and Google Scholar. Search terms included: “salpingitis,” “pelvic inflammatory disease,” “PID,” “hydrosalpinx,” “pyosalpinx,” “tubo-ovarian abscess,” “TOA,” “endometritis,” “adnexitis,” “oophoritis,” and “pelvic peritonitis,” along with their thesaurus equivalents.

We included articles written in English describing pediatric cases (<17 years) diagnosed with salpingitis, hydrosalpinx, pyosalpinx, or TOA. Studies involving sexually active individuals or adult patients were excluded. Articles were screened based on titles and abstracts, followed by full-text review. Discrepancies in inclusion were discussed and resolved by consensus among the authors. Two reviewers (MC and TG) independently screened titles and abstracts and subsequently assessed full texts. Discrepancies were resolved by consensus with a third reviewer (SS). Because the purpose of the review was to contextualize our clinical findings rather than to conduct a systematic assessment of the literature, a narrative review methodology was used and PRISMA guidelines were not applied. Predefined inclusion and exclusion criteria were adopted to maximize consistency and transparency.

## 3. Results

Thirteen patients were diagnosed with salpingitis during the study period; three were sexually active and were excluded (Table 1). The ten non-sexually active girls had a median age of 12.8 years (IQR 2.8), and 4 (40%) were premenarchal. Abdominal pain was the most frequent presenting symptom (80%), followed by fever and gastrointestinal manifestations (50% and 30%, respectively); two patients (20%) were asymptomatic and diagnosed incidentally. Median CRP and white blood cell count at admission were 5.58 mg/dL (IQR 6.21) and 12.095/mm^3^ (IQR 7.270), respectively.

A potential predisposing condition was identified in nine out of ten patients. Among premenarchal girls, factors were predominantly gastrointestinal or abdominal (75%), whereas postmenarchal patients showed gastrointestinal disease, prior pelvic surgery or congenital anomalies, or perimenarchal onset. Overall, gastrointestinal or appendiceal mechanisms accounted for 50% of cases.

Ultrasound was the initial imaging modality in all patients; MRI was performed in eight and CT in two. Hydrosalpinx (60%), pyosalpinx (20%), and nonspecific salpingitis (10%) were the most frequent findings. MRI confirmed all sonographic diagnoses.

A causative organism was identified in three patients (30%): *Citrobacter koseri* and *Escherichia coli* from surgical specimens, and *Gardnerella vaginalis* from a vaginal swab; no sexually transmitted pathogens were detected.

All patients received empiric intravenous broad-spectrum antibiotics, followed by oral therapy. Premenarchal girls had a median hospital stay of 7 days (IQR 3.5–15), and two required surgical management. Postmenarchal girls had a median stay of 11 days (IQR 3–16), with early surgical intervention needed in half of the cases. Overall, 5/10 patients underwent surgery, with no major postoperative complications. At follow-up (available in 8 patients), most showed full clinical recovery.

The literature review identified 41 publications describing 56 non-sexually active pediatric patients with salpingitis or related tubal pathology (Supplementary Table S1). Cases were predominantly postmenarchal (73.2%) and typically presented with abdominal pain; gastrointestinal mechanisms, ascending genitourinary infection, and postsurgical or anatomical conditions were the main predisposing pathways. A causative pathogen was reported in 66.7% of premenarchal and 55.8% of postmenarchal cases, with enteric and anaerobic organisms being the most frequent isolates. Across studies, most patients required surgical management and empiric antibiotics, and TOA emerged as the most common complication. Table 2 provides a comparative analysis of our cohort and previously reported pediatric cases of salpingitis in non-sexually active patients.

## 4. Discussion

Salpingitis in non-sexually active pediatric patients is a poorly defined clinical entity, with current knowledge deriving almost exclusively from isolated case reports and small case series, and no population-level studies are available to date. The absence of a representative pediatric cohort and the lack of standardized diagnostic criteria hinder reliable estimates of prevalence or incidence. Moreover, salpingitis in children is likely underrecognized: it is rare, often not considered in the differential diagnosis, and may present with subtler or atypical features compared to pelvic inflammatory disease in adults. Within this context, our cohort of ten non-sexually active girls represents one of the most substantial pediatric series reported and contributes to refining the clinical profile of this underappreciated condition.

Our findings, in line with existing literature, suggest that salpingitis in non-sexually active pediatric patients represents a distinct clinical entity, characterized by specific patterns of presentation, predisposing factors, and pathogenic mechanisms that differ from those observed in sexually active patients, both adolescents and adults.

Clinical presentation may be subtle; systemic symptoms, particularly fever, were inconsistently reported in both our cohort and published cases, indicating that tubal involvement may occur even in the absence of overt systemic inflammation [7,16,20,21,22,23,24,25,26,27,28,29,30,31,32,33,34,35,36,37,38,39,40,41,42,43,44,45,46,47,48,49,50,51,52,53,54,55,56,57,58,59,60,61].

Abdominal pain was the predominant presenting symptom across pubertal stages, reported in 73% of premenarchal and 83% of postmenarchal patients in published pediatric cases, closely mirroring our cohort [7,16,21,22,23,24,25,26,27,28,29,30,31,32,33,34,35,36,37,38,39,40,41,42,43,44,45,46,47,48,49,50,51,52,53,54,55,56,57,58,59,60,61]. While this consistency underscores abdominal pain as a common initial clinical clue, it has low specificity and substantially overlaps with frequent pediatric abdominal conditions, potentially contributing to delayed recognition.

Gastrointestinal manifestations were also frequent, particularly in premenarchal patients, and showed a similar distribution in our cohort, suggesting a potential role for underlying enteric or appendiceal conditions as associated or predisposing factors in younger girls [7,16,21,22,23,24,25,26,27,28,29,30,31,32,33,34,35,36,37,38,39,40,41,42,43,44,45,46,47,48,49,50,51,52,53,54,55,56,57,58,59,60,61].

Genitourinary symptoms were rare, as expected in non-sexually active girls, with low rates of vaginal discharge reported in the literature (up to 20% in prepubertal and 7% in postmenarchal patients) [7,16,21,22,23,24,25,26,27,28,29,30,31,32,33,34,35,36,37,38,39,40,41,42,43,44,45,46,47,48,49,50,51,52,53,54,55,56,57,58,59,60,61]. Notably, two patients were asymptomatic, consistent with reports suggesting that silent pediatric salpingitis, estimated at approximately 7%, may be underrecognized.

The clear clinical contrast with the three sexually active adolescents in our institution, all of whom presented with vaginal discharge or bleeding (100%) and abdominal pain, highlights the distinct pathophysiological pathways between sexually transmitted and non-sexually transmitted salpingitis. In pediatric salpingitis, gastrointestinal infections, appendiceal disease, and prior abdominal or pelvic surgery were the main predisposing factors, supporting the predominance of non-genital pathways such as enteric translocation, peritoneal spread, or postsurgical anatomical disruption. The published data show the gastrointestinal mechanisms as the leading contributors (40%), followed by genitourinary pathways and pelvic surgical or anatomical abnormalities [7,16,21,22,23,24,25,26,27,28,29,30,31,32,33,34,35,36,37,38,39,40,41,42,43,44,45,46,47,48,49,50,51,52,53,54,55,56,57,58,59,60,61]; see Table 2. Stratified analyses similarly demonstrate a predominantly enteric/translocation-driven etiology in premenarchal girls (40%) and a broader distribution in postmenarchal patients [7,16,21,22,23,24,25,26,27,28,29,30,31,32,33,34,35,36,37,38,39,40,41,42,43,44,45,46,47,48,49,50,51,52,53,54,55,56,57,58,59,60,61] (Table 2).

Microbiological yield was low in our cohort (30%), possibly related to early empiric antibiotic treatment and to the inherent challenges of obtaining optimal microbiological specimens in children. Information on outpatient antibiotic exposure prior to admission was not consistently available due to the retrospective design, which may have contributed to the low microbiological yield. Isolated organisms, *Citrobacter koseri*, *Escherichia coli*, *and Gardnerella vaginalis*, were consistent with non-STI pathways.

In contrast, published pediatric cases report a higher identification rate (67% premenarchal; 56% postmenarchal) [7,16,21,22,23,24,25,26,27,28,29,30,31,32,33,34,35,36,37,38,39,40,41,42,43,44,45,46,47,48,49,50,51,52,53,54,55,56,57,58,59,60,61], although publication bias favoring microbiologically confirmed cases cannot be excluded. These reports reveal a heterogeneous microbial spectrum, predominantly composed of enteric and anaerobic organisms (*Escherichia coli*, *Bacteroides fragilis*, *Fusobacterium nucleatum*), as well as hematogenous pathogens such as *Streptococcus pneumoniae*. A unique case of Neisseria gonorrhoeae transmission in a non-sexually active 5-year-old [40] underscores the importance of considering non-traditional modes of transmission in rare circumstances. Overall, the bacterial ecology in non-sexually active pediatric patients appears distinct from that of STI-driven salpingitis, supporting the central role of enteric, polymicrobial, and occasionally hematogenous mechanisms [3,24,43,44,45,46]. By contrast, all sexually active patients in our institution tested positive for STI pathogens, in line with existing literature [12,20].

Management in our cohort, especially in premenarchal girls, was largely conservative, relying on prolonged antibiotic therapy with selective or delayed surgical intervention. This approach differs from previous reports, where more aggressive surgery, including laparotomy, is common and often linked to complications such as TOA or torsion, likely reflecting a selection bias toward more severe cases in the published literature.

The relatively mild clinical course observed in our patients may be explained by earlier diagnosis and referral, allowing prompt initiation of medical therapy and potentially reducing the need for invasive surgical management, together with a lower prevalence of underlying pelvic disease. The contrast between pre- and postmenarchal patients further suggests a role of pubertal status in disease severity and treatment response.

Early imaging, particularly ultrasound, was crucial for timely diagnosis, supporting its routine use in children presenting with nonspecific abdominal pain.

Strengths of this study include the provision of structured clinical data in an area previously dominated by isolated case reports, helping to delineate diagnostic patterns and risk profiles that may assist clinicians in recognizing salpingitis in non-sexually active pediatric patients. To our knowledge, this represents one of the largest pediatric cohorts reported to date.

Several limitations should also be acknowledged. Despite the cohort size, the study is limited by its modest sample size and single-center design, which restrict generalizability. The absence of universally accepted diagnostic criteria for pediatric salpingitis introduces potential misclassification, particularly given the overlap with other pelvic inflammatory conditions. Microbiological findings were constrained by non-standardized specimen collection and frequent pre-sampling antibiotic exposure. In addition, long-term outcomes, including fertility, recurrence, and tubal function, could not be assessed due to the lack of systematic follow-up, a recurrent limitation in the literature. Finally, the literature review was narrative rather than systematic, which may limit the completeness of case identification. Future multicenter studies with standardized diagnostic frameworks and longitudinal follow-up are needed to clarify pathogenesis, optimize management, and evaluate reproductive outcomes.

## 5. Conclusions

Salpingitis in non-sexually active pediatric patients differs substantially from STI-related disease in terms of clinical presentation, etiologic pathways, and microbiological profiles. Its atypical and often subtle presentation is characterized predominantly by abdominal pain rather than genitourinary symptoms and is frequently associated with gastrointestinal antecedents or prior pelvic surgery, with enteric pathogens commonly implicated. Increased awareness of this entity may reduce diagnostic delays and unnecessary surgical exploration, while facilitating timely initiation of appropriate antibiotic therapy. Management typically involves empiric broad-spectrum intravenous antibiotics, subsequently tailored based on culture and susceptibility results and transitioned to oral therapy, thereby potentially preventing complications, including those that may compromise future fertility.

As current evidence remains limited, coordinated research efforts are needed to establish diagnostic criteria, refine therapeutic strategies, and determine long-term reproductive outcomes.

## Figures and Tables

**Table 1:** Clinical characteristics, management, and outcomes of pediatric salpingitis in a single-center institutional cohort of non–sexually active girls.

	Medical History			Admission				Hospitalization				After Discharge
Non Sexually Active Patients	Age (yr)	Menarche (yr)	Predisposing Condition	Main Symptoms	WBC (Count/ µL)	CRP (mg/dL)	Imaging (Max Size, cm)	Length of Stay	Management	Diagnosis	Pathogen (Exam)	Follow up
1	3.8	None	*Clostridioides difficile* enterocolitis	Palpable abdominal mass	7.730	0.09	US/MRI (8.2 × 5 × 4.5)	10 days	Antibiotic therapy	Left hydrosalpinx	Negative (blood PCR)	Improving at follow-up
2	7.6	None	Immunosuppressive therapy for renal transplant	Abdominal pain, fever	14.970	6.11	US/MRI/CT (9.5)	20 days (surgery day 6)	US-guided drainage + antibiotic therapy	Bilateral pyosalpinx, right TOA	*Citrobacter koseri* (culture from surgical specimen)	Complete resolution (1 month AD)
3	10.9	None	Gastroenteritis	Abdominal pain, vomiting, diarrhea	11.330	7.08	US (Unknown)	3 days	Antibiotic therapy	Right hydrosalpinx	Not mentioned	Complete resolution (1 month AD)
4	11.2	None	Appendicitis with peritonitis	Abdominal pain, dysuria, vaginal blood spotting	12.860	0.26	US/MRI (2.8 × 3.0 × 3.5)	4 days (surgery 1 months after the discharge)	Laparoscopic salpingotomy + Antibiotic therapy	Right pyosalpinx	Negative (blood and urine culture; blood PCR)	Complicated course (salpingectomy at 2 months AD)
5	12.6	12.0	Appendicitis with peritonitis; obesity	Abdominal pain, vomiting, fever	15.000	13.1	US/MRI/CT (2.4 × 3.9 × 3.5)	16 days	Antibiotic therapy	Right salpingitis with TOA	Negative (blood PCR, IGRA)	Persistent disease (3 months AD)
6	13.0	12.9	Recent menarche	Abdominal pain, low back pain	9.850	0.06	US/MRI (5.9 × 3.9 × 2.3)	15 days	Antibiotic therapy	Left hydrosalpinx	*Gardnerella vaginalis* (culture from vaginal swab)	Improving (1 month AD)
7	13.3	12.0	Unknown	Abdominal pain	7.080	5.32	US/MRI (6.2)	3 days	Antibiotic therapy	Left hydrosalpinx	Negative (culture from vaginal swab)	Complete resolution (3 months AD)
8	13.7	13.1	Ovarian torsion in teratoma	Asymptomatic (incidental)	5.096	0.06	US/MRI (Unknown)	3 days (surgery day 2)	Laparoscopic salpingectomy	Right hydrosalpinx	Not mentioned	Complete resolution (1 month AD)
9	13.9	13.1	Prior pelvic surgery	Abdominal pain, vomiting, fever	19.000	5.84	US (Unknown)	7 days (surgery day 2)	Laparoscopic drainage + Antibiotic therapy	Right salpingitis	*Escherichia coli* (culture from surgical specimen)	Interrupted follow-up
10	15.7	Unknown date	Chronic appendicitis	Abdominal pain, fever	22.300	6.30	US/MRI (Unknown)	21 days (surgery 2 months after the discharge)	Antibiotic therapy	Bilateral salpingitis	Negative (blood PCR, urinoculture)	Complicated course; additional surgery at 2 months AD

AD = after discharge; CRP = C-reactive protein; CT = computed tomography; MRI = magnetic resonance imaging; PCR = polymerase chain reaction; IGRA = Interferon-Gamma Release Assay; TOA = tubo-ovarian abscess; US = ultrasound; WBC = white blood cell count.

**Table 2:** Comparison between our cohort and published pediatric cases of salpingitis, stratified by sexual activity and pubertal status, including presenting symptoms, identified pathogens, and suspected etiologies.

	Cases from Our Hospital (*n* = 10)			Published Cases (*n* = 56)		
Pubertal Status	Onset Symptoms	Pathogens	Suspected Etiology	Onset Symptoms	Pathogens	Suspected Etiology
**Pre pubertal**	Abdominal pain (75%) Fever (25%) Vomiting (25%) Bowel-habits changes (25%) Dysuria (25%) Vaginal spotting (25%) Asymptomatic (25%)	*Citrobacter koseri* (25%) Unknown (75%)	Non-surgical gastrointestinal conditions (50%) Past abdominal surgery (25%) Immunosuppression (25%)	Abdominal pain (73.3%) Vomiting (46.7%) Fever (33.3%) Vaginal discharge (20%) Dysuria (6.6%) Bowel-habits changes (6.6%) Asymptomatic (13.3)	*Streptococcus* spp. (40%) *Escherichia coli* (13.3%) *Bacteroides fragilis* (13.3%) *Fusobacterium* spp. (13.3%) *Neisseria gonorrhoeae* (13.3%) *Proteus mirabilis* (13.3%)	Non-surgical gastrointestinal conditions (46.7%) Hematogenous spread (20%) Past abdominal surgery (6.7%) Immunosuppression (6.7%) UTIs (6.7%)
**Post menarche**	Abdominal pain (83.3%) Fever (50%) Vomiting (33.3%) Asymptomatic (16.7%)	*Gardnerella vaginalis* (16.7%) *Escherichia coli* (16.7%) Unknown (66.7%)	Past abdominal surgery (30%) Non-surgical gastrointestinal conditions (10%) UTIs (10%) Obesity (10%) Menarche (10%) Hematogenous spread (10%) Malformation (10%) Unknown (10%)	Abdominal pain (82.9%) Fever (39.0%) Vomiting (26.8%) Vaginal discharge (7.3%) Bowel-habit changes (9.8%) Dysuria (9.8%) Dyspnea (2.4%) Asymptomatic/incidental (7.3%)	*Escherichia coli* (31.7%) *Streptococcus* spp. (29.3%) *Staphylococcus* spp. (9.8%) *Bacteroides fragilis* (7.3%) *Prevotella* spp. (4.9%) *Fusobacterium* spp. (2.4%)	Non-surgical gastrointestinal conditions (43.9%) Past abdominal surgery (36.6%) UTIs (14.6%) Hematogenous spread (7.3%) Malformations (7.3%) Obesity (7.3%) Immunosuppression (4.9%)
**Overall**	Abdominal pain 80% Fever (50%) Vomiting (30%) Asymptomatic (20%)	*Citrobacter koseri* (10%) *Gardnerella vaginalis* (10%) *Escherichia coli* (10%) Unknown (70%)	Past abdominal surgery (40%) Non-surgical gastrointestinal conditions (30%) UTIs (10%) Obesity (10%) Immunosuppression (10%) Menarche (10%) Hematogenous spread (10%) Malformation (10%) Unknown (10%)	Abdominal pain (80.4%) Fever (37.5%) Vomiting (32.1%) Vaginal discharge (10.7%) Bowel-habits changes (8.9%) Dysuria (8.9%) Asymptomatic (7.1%)	*Escherichia coli* (52.2%) *Streptococcus* spp. (32.1%) *Staphylococcus* spp. (9.8%) *Bacteroides* (7.4%) *Prevotella* spp. (3.6%) *Fusobacterium* spp. (3.6%) *Neisseria gonorrhoeae* (1.8%) *Proteus mirabilis* (1.8%)	Non-surgical gastrointestinal conditions (39.3%) Past abdominal surgery (33.9%) UTIs (20.7%) Hematogenous spread (20.7%) Immunosuppression (5.4%) Malformations (5.4%) Obesity (5.4%)

## Data Availability

The data presented in this study are available on request from the corresponding author. The data are not publicly available due to privacy and ethical restrictions.

## Supplementary Materials

The following supporting information can be downloaded at: https://www.mdpi.com/article/10.3390/children13030311/s1, Table S1: Salpingitis in non–sexually active pediatric patients identified through a literature review.
