# Supplementary material for: Salpingitis in Non-Sexually Active Girls: Clinical Spectrum and Diagnostic Clues from a Pediatric Cohort
Source: Children (Basel). 2026 Feb 24;13(3):311. doi: 10.3390/children13030311 (PMC13025358; doi:10.3390/children13030311)
Supplement: Supplementary file 1 [file children-13-00311-s001.zip › children-4093984-Supplementary Table S1 Clean- literature review.pdf]

Supplementary Table S1. Salpingitis in non–sexually active pediatric patients identified through a literature review.

Reference	Age (years)	Pubertal status	Symptoms	Diagnosis	Length of stay (days)	Pathogen	Therapy	Complications	Predisposing factors
Claireux et al. [41]	0	Premenarchal	Newborn with autoptic finding of pyosalpinx	Right pyosalpinx	32 days	*Balantidium coli, Bacillus proteus,* non–group A *Streptococcus* (vaginal discharge)	Empiric antibiotics	Death	Neonatal sepsis from mother hepatitis
Rossback et al. [42]	3	Premenarchal	Acute abdomen	Salpingitis	Unknown	Not mentioned	Laparoscopic drainage	None	Typhlitis 3-years before in a patient with acute lymphoblastic leukemia
Meis et al. [43]	4	Premenarchal	Upper abdominal pain, nausea and vomiting	Salpingitis	Unknown	*Streptococcus pneumoniae*	Unknown	Unknown	Previous abdominal laparotomic surgery for trauma, hematogenous translocation
Burke et al. [44]	5	Premenarchal	Abdominal pain, vaginal discharge and fever	Salpingitis	Unknown	Unspecified Gram-negative diplococci	Unknown	Unknown	Unknown
Auman et al. [40]	5	Premenarchal	Thick and non-pruritic vaginal discharge, fever, vomiting	Right salpingitis	5 days	*Neisseria gonorrhea* (vaginal discharge)	Laparoscopic surgery, empiric and targeted antibiotics	None	Sleeping with 16-years sexually active cousin with vaginal discharge
Touloukian et al. [26]	6	Premenarchal	Abdominal pain, fever, bile-stained vomiting and vaginal discharge	Salpingitis	Unknown	*Escherichia coli*	Unknown	None	Cerebral palsy, urine and stool incontinence: bacterial translocation
Fei YF et al. [22]	7	Premenarchal	Asymptomatic. Incidentaloma during nephrological checks for past renal transplant	Salpingitis	8 days	Negative	Laparotomic surgery and empiric antibiotics	Tubo-Ovarian Abscess (TOA)	Immunosuppressive therapy and bacterial translocation
Sirotnak AP et al. [45]	8	Premenarchal	Three days of abdominal pain, nausea and fever	Right pyosalpinx	Unknown	*Streptococcus pneumoniae* serotype 1	Laparoscopic surgery, empiric antibiotics	None	Hematogenous spread
Habek D et al [46]	9	Premenarchal	Acute abdomen	Right pyosalpinx	Unknown	*Escherichia coli* (vaginal discharge and fallopian pus culture)	Laparotomic surgery and empiric antibiotic	None	Ascending infection from the lower genital tract causing pyosalpinx and lately peritonitis
Fei YF et al. [22]	10	Premenarchal	Urinary and fecal incontinence and constipation	Salpingitis	15 days	Negative	Empiric antibiotics, exploratory laparoscopy	TOA	Bacterial translocation
Kielly M et al. [2]	11	Premenarchal	Acute onset abdominal pain, vomiting and nausea	Bilateral salpingitis	14 days	Negative	Laparoscopic drainage and empiric antibiotics	Non	Long history of functional constipation and occasional encopresis causing bacterial translocation
Van der Putten ME et al. [47]	11	Postmenarchal	Diffuse abdominal pain, fever, nausea	Right salpingitis	10 days	*Streptococcus pneumoniae* (fallopian fibrin culture and blood culture)	Exploratory laparoscopy and empiric antibiotics	None	Hematogenous spread from upper airways infection
Merlini L et al. [27]	11	Postmenarchal	Asymptomatic. US-incidentaloma on annual follow-up controls for Wilms tumor	Bilateral hydrosalpinx	Unknown	Negative	None	None	Postsurgical complication (Hirschsprung disease, Wilms tumour), constipation (congenital defect of the autonomous innervation)
Sirotnak AP et al. [45]	12	Premenarchal	Right lower abdominal pain, vomiting	Right salpingitis	Unknown	*Streptococcus pneumoniae* serotype 1	Laparoscopic surgery, empiric antibiotics	TOA	Hematogenous spread
Sirotnak AP et al. [45]	12	Postmenarchal	Crampy abdominal pain lasting for 2 days and difficult breathing	Bilateral salpingitis	Unknown	*Streptococcus pneumoniae* serotype 1	Laparoscopic surgery, empiric antibiotics	None	Hematogenous spread

Barak G et al. [21]	12	Postmenarchal	Acute onset of right lower quadrant abdominal pain	Bilateral hydrosalpinx	Unknown	Escherichia coli (from urinary culture)	Empiric antibiotics	None	Constipation as cause of urinary stasis and ascending UTI
Maraqa T et al. [35]	12	Postmenarchal	Abdominal pain	Bilateral pyosalpinx		Peptostreptococcus anaerobius, Prevotella bivia, S. anginosus (surgical sample culture)	Exploratory laparoscopy, empirical antibiotics	None	Past histories of recurrent UTI, irritable bowel syndrome and recent diagnosis of obstructed hemivagina (Mullerian duct anomaly)
Desai B et al. [29]	12	Postmenarchal	Abdominal pain and watery vaginal discharge	Bilateral pyosalpinx	10 days	Not mentioned	Empirical antibiotics	None	History of Hirschsprung's disease with a staged repair
Merlini L et al. [27]	12	Postmenarchal	Two-days history of acute right pelvic pain	Right salpingitis	Unknown	Not mentioned	Laparoscopic surgery	None	Past surgery for Hirschsprung disease, congenital defect of the autonomous innervation, concomitant appendicitis
Azumagawa K et al. [23]	12	Postmenarchal	Muddy diarrhea, frequent vomiting, and severe abdominal pain	Bilateral hydrosalpinx	Unknown	Escherichia coli (surgical sample culture)	Exploratory laparoscopy, empirical antibiotics	TOA	Bacterial fecal translocation, obesity (BMI 31)
Hartmann KA et al. [48]	12	Postmenarchal	One-day history of diffuse lower abdomen pain, nausea, vomiting, and a fever	Bilateral salpingitis	Unknown	Escherichia coli (abscess cultures)	Empiric antibiotics	TOA, persistence of hydrosalpinx	Obesity, constipation, recurrent urinary tract infections, poor hygiene
Nishida N et al. [32]	12	Postmenarchal	Fever and right lower quadrant pain	Salpingitis	6 days	Negative	Exploratory laparoscopy, empirical antibiotics	TOA	Severe pelvic adhesions due to previous perforated appendicitis
Fei YF et al. [22]	12	Postmenarchal	Abdominal pain	Salpingitis	13 days	Prevotella, Peptococcus magnus, Streptococcus anginosus	Laparotomy, empiric antibiotics	TOA	Cervical-vaginal atresia and VACTERL sequence
Fei YF et al. [22]	12	Postmenarchal	Abdominal pain	Salpingitis	3 days	Not collected	Empiric antibiotics	TOA	Obesity (BMI 41), nocturnal enuresis, poor hygiene
Fein D et al. [49]	12	Postmenarchal	Right lower abdominal pain and recent fever and dysuria	Bilateral salpingitis	7 days	Not collected	Exploratory laparoscopy, empiric antibiotic	None	Previous IVU, surgical appendicectomy 2 years before
Moralioğlu S et al. [28]	13	Postmenarchal	Abdominal pain	Hydrosalpinx	None	Not mentioned	Exploratory laparoscopy	None	Hirschsprung disease (innervation defects), surgical adherence, fecal incontinence
Pampal A. et al. [50]	13	Postmenarchal	Lower left abdominal pain lasting 2 days, nausea and vomiting	Hematosalpinx	3 days	Not mentioned	Laparotomic surgery	Ovarian torsion	Unknown
Hornemann a et al. [51]	13	Postmenarchal	Right lower abdominal pain 2-days long, nausea, fever	Right pyosalpinx	5 days	Escherichia Coli (surgical material)	Laparoscopic surgery and empiric antibiotics	None	Unknown
Murata et al. [52]	13	Postmenarchal	One-month lasting fever without any other symptoms	Right pyosalpinx	Unknown	Staphylococcus aureus (abscess culture)	Laparoscopic surgery and empiric antibiotics	TOA	Unknown source of infection, suspected bloodstream
Lima M et al. [53]	13	Premenarchal	Lower abdominal quadrants pain	Bilateral hydrosalpinx	Unknown	Not mentioned	Laparoscopic surgery	Ovarian torsion	Unknown
Hornemann a et al. [51]	13	Postmenarchal	Upper quadrant abdominal pain and fever	Right pyosalpinx	5 days	Escherichia coli (surgical material)	Laparoscopic surgery and empiric antibiotics	None	Unknown
Sakar MN et al. [54]	13	Postmenarchal	Abdominal pain and menstrual disorder	Salpingitis	Unknown	Not mentioned	Laparoscopic surgery and empiric antibiotics	TOA	Ascending infection from the lower genital tract
Fei YF et al. [22]	13	Premenarchal	Fever, abdominal pain, dysuria	Salpingitis	9 days	Bacteroides fragilis	Laparoscopic surgery, empiric and targeted antibiotics	TOA	UTIs, ascending infection or translocation from the lower genital tract

Fumino et al. [55]	13	Postmenarchal	Asymptomatic. Incidentaloma during radiological follow up checks	Salpingitis	Unknown	Not mentioned	Laparoscopic surgery and empiric antibiotics	TOA	Vaginoplasty (congenital cloaca anomaly), adherences, bacterial translocation
Goodwin K et al. [56]	13	Postmenarchal	Colicky abdominal pain and vomiting and constipation	Salpingitis	14 days	Escherichia coli (abscess cultures)	Laparoscopic surgery and empiric antibiotics	TOA	Previous appendicectomy, constipation
Fei YF et al. [22]	13	Postmenarchal	Vaginal discharge, abdominal pain	Salpingitis	8 days	Bacteroides fragilis, Peptostreptococcus anaerobius	Laparotomic surgery and empiric antibiotics	TOA	Foreign body-induced inflammatory reaction (foreign body placed two years before in vagina), bacterial translocation
Cheong LHA et al. [57]	13	Postmenarchal	Fever, anorexia, vomiting, and fluctuating abdominal pain	Pyosalpinx	7 days	Streptococcus viridans, Peptostreptococcus (abscess culture)	Laparotomic surgery and empiric antibiotics	TOA	Unknown
Mills D et al. [25]	13	Postmenarchal	Abdominal pain	Bilateral salpingitis	Unknown	Streptococcus constellatus	Laparoscopic surgery and empiric antibiotics	TOA	Past interventional US-guided drainage of an appendiceal abscess, bacterial translocation from chronic appendicitis
Simpson-Camp L et al. [58]	14	Postmenarchal	Fatigue and low-grade fever	Pyosalpinx	14 days	Streptococcus viridans (surgical drainage)	Laparoscopic surgery, empiric and targeted antibiotics	TOA	Ascending infection from the lower genital tract, poor hygiene
Stortini et al. [36]	14	Postmenarchal	Acute urinary retention, fever	Bilateral pyosalpinx	14 days	S. anginosus, Peptostreptococcus anaerobius (surgical drainage)	Laparoscopic surgery, empiric and targeted antibiotics	TOA	Ascending infection from pooling of urine in the vagina due to labial agglutination caused by lichen sclerosus, recurrent IVU
Moralioğlu S et al. [28]	14	Postmenarchal	Abdominal pain, vomiting and fever	Right pyosalpinx	Unknown	Escherichia coli (pus culture)	Laparoscopic drainage, antibiotic therapy	None	Congenital anomaly, pelvic surgery (recto-vestibular fistula with anal atresia), adhesions, fecal bacteria translocations
Scarpa et al. [59]	14	Postmenarchal	Lower quadrant acute pain	Right hydrosalpinx	Unknown	Not mentioned	Empiric antibiotic therapy	Bilateral hydrosalpinx	Steroid therapy required by Congenital Adrenal Hyperplasia (CAH)
Algren SD et al. [60]	14	Premenarchal	Lower abdominal pain, fever, dysuria, night sweats, nausea, vomiting, diarrhea	Salpingitis	Unknown	Streptococcus, Fusobacterium nucleatum	Unknown	None	Unknown
Scarpa et al. [59]	15	Postmenarchal	Abdominal pain, fever	Bilateral pyosalpinx	14 days	Not mentioned	Empiric antibiotic therapy	None	Steroid therapy required by CAH, past surgical treatment for vaginal stenosis
Moore MM et al. [61]	15	Postmenarchal	Abdominal pain, nausea and vomiting, fever, dysuria	Salpingitis	17 days	Escherichia coli (abscess culture)	Laparoscopic surgery, empiric antibiotics	TOA	Urinary bacterial translocation (previous recurrent cystitis), recessed urethra
Arda IS et al. [37]	15	Postmenarchal	Abdominal pain, dysuria, fever	Salpingitis	Unknown	Escherichia coli (urine culture)	Empiric and targeted antibiotics	None	Combination of concomitant UTI and previous appendicectomy
Fei YF et al. [22]	15	Postmenarchal	Abdominal pain	Salpingitis	5 days	Not collected	Empiric antibiotics	TOA	History of open appendicectomy, abdominal bacterial translocation
Fei YF et al. [22]	15	Postmenarchal	Constipation	Salpingitis	2 days	Not collected	Empiric antibiotics	TOA	Abdominal bacterial translocation
Rubino C et al. [3]	15	Postmenarchal	Tender abdomen, hyperemic external genitalia, and profuse leucorrhea	Recurrent bilateral salpingitis	21 days	Negative	Empiric antibiotic	None	Bacterial translocation from chronic appendicitis.
Kielly M et al. [2]	15	Postmenarchal	General malaise, perfuse diarrhea and right lower quadrant pain	Salpingitis	14 days	Negative	Laparoscopic surgery, empiric antibiotics	None	Unknown

Boleken ME et al. [16]	15	Postmenarchal	Left lower quadrant pain lasting for a week	Xanto-granulomatous salpingitis	9 days	Escherichia coli (surgical culture)	Laparotomic surgery, empiric and targeted antibiotic	None	Bacterial translocation with subsequent xanthogranulomatous salpingitis due to chronic constipation
Limberg J et al. [30]	15	Postmenarchal	Recurrent right lower quadrant/pelvic abscess	Ruptured right hydrosalpinx	Unknown	Streptococcus anginosus (surgical drainage)	Interventional radiology and antibiotic therapy	Chronic follicular salpingitis	Perforated appendicitis (3 years before)
Niyogi A et al. [31]	15	Postmenarchal	Abdominal pain, fever, vomiting, nausea 5-days lasting	Recurrent unilateral hydrosalpinx	Unknown	Not mentioned	Surgical drainage	None	Previous necrotic perforated appendicitis
Hartmann KA et al. [48]	16	Postmenarchal	Three-week history of suprapubic and right lower quadrant abdominal pain, anorexia, fever	Right salpingitis		Bacteroides uniformis, Staphylococcus, Streptococcus milleri (abscess cultures)	Exploratory laparoscopy	TOA	Crohn's disease onset sign
Fei YF et al. [22]	16	Postmenarchal	Unknown	Salpingitis	7 days	Escherichia coli	Exploratory laparoscopy	TOA	Unknown
Merlini L et al. [27]	16	Postmenarchal	None. Incidentaloma on pre-operative US for nonobstructive cribriform hymen	Bilateral hydrosalpinx	None	Not mentioned	None	None	Previous surgery for Hirschsprung disease
Takeda M et al. [33]	17	Postmenarchal	Left lower quadrant pain found	Left hydrosalpinx		Not mentioned	Laparoscopic surgery	Tubal torsion	Previous left hernioplasty (adhesions causing blockage of fluid drainage)
Singh-Ranger D et al. [24]	17	Postmenarchal	Lower abdominal and back pain 2-days lasting	Pyosalpinx	8 days	E. coli (intraoperative fallopian pus culture)	Surgical drainage and empiric antibiotics		Appendectomy for gangrenous appendicitis three months before

BMI = body mass index; TOA = tubo-ovarian abscess; UTI = urinary tract infection.
